# A multiyear systematic survey of the quality of reporting for randomised trials in dentistry, neurology and geriatrics published in journals of Spain and Latin America

**DOI:** 10.1186/s12874-021-01337-3

**Published:** 2021-07-26

**Authors:** Vivienne C. Bachelet, María S. Navarrete, Constanza Barrera-Riquelme, Víctor A. Carrasco, Matías Dallaserra, Rubén A. Díaz, Álvaro A. Ibarra, Francisca J. Lizana, Nicolás Meza-Ducaud, Macarena G. Saavedra, Camila Tapia-Davegno, Alonso F. Vergara, Julio Villanueva

**Affiliations:** 1 Escuela de Medicina, Facultad de Ciencias Médicas, Universidad de Santiago de Chile (USACH), Avenida Libertador Bernardo OHiggins 3363, Santiago, Estación Central Chile; 2 Departamento de Cirugía Maxilofacial, Facultad de Odontología, Universidad de Chile, Santiago, Chile; 3 Hospital Clínico San Borja-Arriarán, Santiago, Chile

## Abstract

**Background:**

The Iberoamerican Cochrane Network is currently developing an extensive project to identify Spanish-language journals that publish original clinical research in Spain and Latin America. The project is called BADERI (Database of Iberoamerican Essays and Journal) and feeds the research articles, mainly randomised clinical trials (RCTs), into CENTRAL (Cochrane Collaboration Central Register of Controlled Trials). This study aims to assess the quality of reporting of RCTs published in Spanish and Latin American journals for three clinical fields and assess changes over time.

**Methods:**

We did a systematic survey with time trend analysis of RCTs for dentistry, geriatrics, and neurology. These fields were chosen for pragmatic reasons as they had not yet been completed in BADERI. After screening RCTs from 1990 to 2018 for randomised or quasi-randomised clinical trials, we extracted data for 23 CONSORT items. The primary outcome was the total score of the 23 predefined CONSORT 2010 items for each RCT (score range from 0 to 34). The secondary outcome measure was the score for each one of these 23 items.

**Results:**

A total of 392 articles from 1990 to 2018 were included as follows: dentistry (282), neurology (80), and geriatrics (30). We found that the overall compliance score for the CONSORT items included in this study for all 392 RCTs analysed was 12.6 on a scale with a maximum score of 34. With time, the quality of reporting improved slightly for all RCTs. None of the articles achieved the complete individual CONSORT item compliance score. The lowest overall compliance percentage was for item 10 (Randomisation implementation) and item 24 (Protocol registration), with a dismal 1% compliance across all included RCTs, regardless of country.

**Conclusions:**

CONSORT compliance is very poor in the 392 analysed RCTs. The impact of the CONSORT statement on improving the completeness of RCT reporting in Latin America and Spain is not clear. Iberoamerican journals should become more involved in endorsing and enforcing adherence to the CONSORT guidelines.

## Main ideas

- This is the first study to conduct a systematic survey of reporting quality of randomised clinical trials for more than one clinical field.
- Our results are consistent with the findings of similar studies in the same clinical fields from other settings.
- This study's main limitations lie in the non-inclusion of RCTs from Brazil, the absence of a standardised CONSORT adherence extraction form designed for quality of reporting control, and the large team of reviewers that could affect the consistency of extraction.
- More efforts must be made directly with journal editors to improve article compliance with CONSORT guidelines.

## Introduction

Randomised clinical trials (RCTs) are considered the best study design to evaluate the effects, benefits, and harms of therapeutic interventions, drugs, devices, or techniques in humans [1, 2]. RCTs are also the basis for systematic reviews and other evidence synthesis documents [3–5]. However, the identification of all RCTs for a given intervention is challenging due to publication bias problems [6].

The quality of the RCTs depends on their internal validity, which results from their methodology, design, and execution. Hence, the completeness of the published information for each component of an RCT is essential to assess internal validity. A well-designed and reported study helps researchers and editors evaluate the study's quality and ensures its correct indexing in the databases. The information from an RCT should be accurate and transparent so that medical professionals can fully assess the quality and methodological rigour, resulting in better-informed decisions [7]. The CONSORT statement (Consolidated Standards of Reporting Trials) was developed in 1996 and updated in 2010 to its present form to improve RCTs' reporting of methods and findings [8].

Several authors have previously pointed out that using electronic indexing services only, such as PubMed or EMBASE, has proven to be suboptimal for RCT identification for several reasons [9–11]. Firstly, the term 'RCT' was not indexed until 1990 and was incorporated into MEDLINE and EMBASE in 1991 and 1994, respectively. Therefore, RCTs before this date were classified using broader categories, such as 'clinical trial', 'controlled trial', 'experimental study', 'randomized controlled clinical trial', or, simply, 'trial'. Secondly, the data suggest that the database descriptors have been used inconsistently by those responsible for their coding and classification [12], leading to more recent efforts to improve searchability with MeSH terms [13–15]. Thirdly, on some occasions, authors do not report their research methods transparently and accurately, making the indexing process of RCTs cumbersome [16]. To address this, the Cochrane Collaboration has recommended hand searching in a selected journal to identify studies that potentially address the research question. In 2007, Hopewell et al. included 34 studies comparing the number of trials identified by manual searching versus those identified by electronic searches [10]. These authors could locate only 49% to 67% of the RCTs in the consulted electronic databases. Furthermore, they found that the recovery rate for an electronic search was lower when the search was restricted to languages ​​other than English, 39% versus 62%. It has also been pointed out that excluding non-English language studies can contribute to language bias [17] and that methods should be sought out to facilitate retrieval and reading of non-English language studies [18]. However, the consequences of not including other languages in systematic reviews are still unclear, with contradictory conclusions [19–22].

To reduce language bias, the Iberoamerican Cochrane Network is currently developing an extensive project to identify Spanish-language journals that publish original clinical research in Spain and Latin America. Within these journals, the Network is conducting manual searches to retrieve all RCTs published in Spain and Latin America by speciality. A database was built on an internet platform to coordinate manual search activities for RCTs. The database is called BADERI (Database of Iberoamerican Essays and Journal) and is integrated into CENTRAL (Cochrane Collaboration Central Register of Controlled Trials) to feed into systematic reviews carried out by the Cochrane Collaboration on different health issues. BADERI was officially launched in October 2015, and the methods used in the identification and inclusion process of RCTs in the database have been previously described [23]. Until 2017, it had included 6583 references to RCTs published in more than 400 journals of Spain and Latin America, related to 46 medical specialities, covering a period between 1957 and 2018. There are publications on the manual searches for RCTs in the fields of obstetrics and gynaecology [24], physiotherapy [25], ophthalmology [26], orthopaedics and traumatology [27], dentistry [28], and dermatology [29]. Several of these reports also include a risk of bias assessment.

The extensive assessment of clinical trials published in the Iberoamerican region should assist editors, evaluators of research funds, and clinical investigator communities in improving their decisions on the planning, execution, and reporting of RCTs [30]. This article presents the results of the previously published protocol for this study [31].

The purpose of this research project is to complete the BADERI database of RCTs in dentistry, neurology, and geriatrics of the Cochrane Collaboration by hand searching and, for these clinical fields, to assess the quality of reporting. This assessment gives us an overall albeit indirect view of the methodological quality of RCTs published in Spanish and Latin American journals. The secondary objectives are to characterise RCTs published in journals from Spain and Latin America included in the Cochrane BADERI database for these three clinical fields and to assess the association between quality of reporting and year of publication vis à vis the latest CONSORT statement.

## Methods

### Design

We did a systematic survey of the BADERI-included RCTs for dentistry, geriatrics, and neurology, with time trend analysis. We chose these specialities for pragmatic reasons as they had a higher number of studies already identified in the BADERI database and had not been previously analysed nor the results published. The full study methods are described in the published protocol [31]. In this article, we summarise the methods used and report deviations from the published protocol, if any.

### Data sources and study selection

We used the RCTs identified via hand searching of Spanish and Latin American journals for dentistry, neurology, and geriatrics as registered in the BADERI database. The BADERI database was updated to 2017, so we closed the gap to the end of 2018 by identifying any missing RCTs for those specialities. By the end of 2019, no new journals had been identified beyond those that BADERI had already registered.

We screened all the included RCTs from 1990 to 2018, applying the inclusion criteria established in our protocol: randomised or quasi-randomised clinical trials with a recoverable full text reporting the full results. Quasi-randomised trials were included following BADERI and Cochrane guidance on the inclusion of clinical trials in Cochrane CENTRAL [32, 33].

### Data extraction

We extracted data for 23 CONSORT items, plus four additional items not analysed in this paper. The CONSORT items included were the following: 1a, 1b, 3a, 4a, 4b, 5, 6a, 7a, 8a, 9, 10, 11a, 12a, 13a, 13b, 14a, 15, 16, 17a, 19, 23, 24, and 25. Table 1 shows the details for these items.

**Table 1:** The instrument used to extract data on CONSORT variables (items)

CONSORT item	#	Description and definition	Scoring
Title	1a	Identification as a randomised trial in the title	1 = word random appears in the title 0 = no word "random" appears
Abstract	1b	Structured summary of trial design, methods, results, and conclusions (for specific guidance see CONSORT for abstracts)	1 = Structured abstract 0 = No structured abstract
Trial design	3a	Description of trial design (such as parallel, factorial) including allocation ratio (ex-split-mouth)	1 = well described design 0 = not well-described design
Participants	4a	Eligibility criteria for participants	1 = specified 0 = not specified
	4b	Settings (A) and locations (B) where the data were collected	2 = A and B 1 = A or B 0 = not specified
Interventions	5	The interventions for each group (A) with sufficient details to allow replication, including how and when they were actually administered (B) (i.e. "usual care" for control group not enough)]	2 = A and B 1 = A, but only one group with details 0 = only A or only one group without details
Outcomes	6a	Completely defined pre-specified primary (A) and secondary outcome (B) measures, including how and when they were assessed (C)	2 = A and B and C 1 = A or B (no distinction) + C 0 = A or B, no C
Sample size	7a	How sample size was determined	1 = specified 0 = not specified
Sequence generation	8a	The method used to generate the random allocation sequence	1 = specified 0 = not specified
Allocation concealment mechanism	9	The mechanism used to implement the random allocation sequence (such as sequentially numbered containers), describing any steps taken to conceal the sequence until interventions were assigned	1 = steps for concealment specified 0 = concealment not specified
Implementation	10	Who generated the random allocation sequence (A), who enrolled participants (B), and who assigned participants to interventions (C)	2 = A and B and C 1 = (A and B) or (A and C) 0 = A missing
Blinding	11a	If done, who was blinded (A) after assignment to interventions (for example, participants, care providers, those assessing outcomes) and how (B)	2 = (A and B) OR reason why the study is open label 1 = declares who is blinded but no details as how 0 = declares the study blind but no who nor how
Statistical methods	12a	Statistical methods used to compare groups for primary and secondary outcomes	2 = states full stats method for each outcome 1 = states stats methods for primary outcome 0 = states stats methods vaguely
Participant flow (a diagram is strongly recommended)	13a	For each group, the numbers of participants who were randomly assigned (A), received intended treatment (B) and were analysed for the primary outcome (C)	2 = A and B and C (narrative in text OR complete flow diagram) 1 = A or B or C missing (only one missing) 0 = only one reported or no info at all
	13b	For each group, losses (A) and exclusions (B) after randomisation, together with reasons (C)	2 = A and B and C 1 = A or B or C missing (only one missing) 0 = only one reported, or no info at all
Recruitment	14a	Dates defining the periods of recruitment (A) and follow-up (B)	2 = A and B reported 1 = A or B reported 0 = none reported
Baseline data	15	A table showing baseline demographic and clinical characteristics for each group	1 = "Table 1" present 0 = "Table 1" not present
Numbers analysed	16	For each group, number of participants (denominator) included in each analysis (A) and whether the analysis was by original assigned groups (B) [ITT or Per Protocol]	2 = A and B 1 = A or B 0 = not stated
Outcomes and estimation	17a	For each primary and secondary outcome, **results** for each group (A), and the estimated **effect size** (B) and the **precision** (confidence interval) (C) [only for primary outcome]	2 = A and B and C 1 = A or B or C missing (only one missing) 0 = only one reported or none
Harms	19	All important harms or unintended effects in each group	1 = harms described 0 = harms not described
Registration	23	Registration number and name of **trial registry**	1 = present 0 = absent
Protocol	24	Where the full trial **protocol** can be accessed, if available	1 = present 0 = absent
Funding	25	Sources of **funding** and other support (such as the supply of drugs)	1 = present 0 = absent
Additional item	AIa	Total number of patients randomised	Annotate sample size
Additional item	AIb	Conflict of interest statement	1 = present 0 = absent
Additional item	AIc	Ethics review	1 = present 0 = absent
Additional item	AId	Language of article	Spanish/English/Portuguese

We obtained the country of publication, but we did not obtain other country data as specified by the protocol because we did not deem it relevant to conduct such an analysis given the similarity of Latin American countries. We extracted the impact factor for the whole list of Latin American and Spanish journals.

After a period of training and calibration, we did a pilot run-in until there was less than 20% discrepancies between reviewers on the same set of assigned articles, differing from our published protocol where we had aimed for 10%. All data extraction for dentistry was done independently by five pairs of reviewers in parallel (AFV-FJL; FJL-NMD, RAD-NMD, RAD-VAC, VAC-AFV). Data extraction for geriatrics and neurology was done independently by four pairs of reviewers in parallel (AAI-CTD, AAI-CBR, CTD-MGS, CBR-MGS). MSN identified the discrepancies and notified the reviewers using an online project-control platform. Accordingly, each pair of reviewers resolved the discrepancies internally by consensus. When consensus was not possible, MSN made the final decision. MD checked 10% of randomly selected papers to assess agreement with previous data extraction from the reviewers. Concordance was adequate, at 80%. Data extraction was done between February and May of 2020.

### Outcomes

In this paper, we report the total score for the 23 predefined CONSORT 2010 items for each RCT (score range from 0 to 34) (Table 1), which provides an overview of each article's overall compliance. Using this overall outcome, we make comparisons over time and before and after the CONSORT 2010 implementation. The secondary outcome measure was the score for each item of the 23 predefined CONSORT items across articles and clinical fields.

Each item was measured either as a binary outcome (yes/no) or with three ordinal categories (full reporting, partial reporting, no reporting). The three-level items were converted into binary variables to facilitate comparisons with other similar studies. We excluded items reporting on Introduction and Discussion due to the inherent subjectivity in the appraisal of these items (2a, 2b, 20, 21, and 22). We also disregarded items 3b, 4b, 6b, 7b, 8b, 11b, 12b, 14b, 17b, and 18 because of potential non-applicability. The resulting score, consequently, ranges from 0 to 34 on the included CONSORT items. We also extracted data on the total number of patients recruited, conflict of interest statement (present/absent), ethics review (present/absent), the language of publication (Spanish, English, Portuguese), and year and country of publication. Funding, conflicts of interest, and ethics approval are not reported in this paper.

### Statistical analysis

Only a descriptive analysis with summary statistics was done given that we included all the RCTs for each clinical field; hence, no statistical inference techniques were necessary. The primary analysis was to compute the mean scores found on the articles by country and by field, and differences were analysed for articles published before 2010 (year included) and articles published after 2011 (year included). The secondary analysis describes each of the 23 CONSORT items included in the study to determine compliance and explore those that most contribute to non-compliance. We provide charts with percentages of RCTs complying with each item, by year and by periods (pre-CONSORT and post-CONSORT 2010). We explored changes for the primary and secondary outcomes by extracting sample size, country of publication, language, and whether the RCTs were published in Spanish journals or Latin American journals.

We used the R package statistical software (R Foundation for Statistical Computing, Vienna, Austria; 2019) for analysis.

### Ethics

The Institutional Ethics Committee of the University of Santiago of Chile approved this study, report No. 524, dated 15 August 2018. No patients were involved.

## Results

### Description of the population of included RCTs

**Fig. 1:**
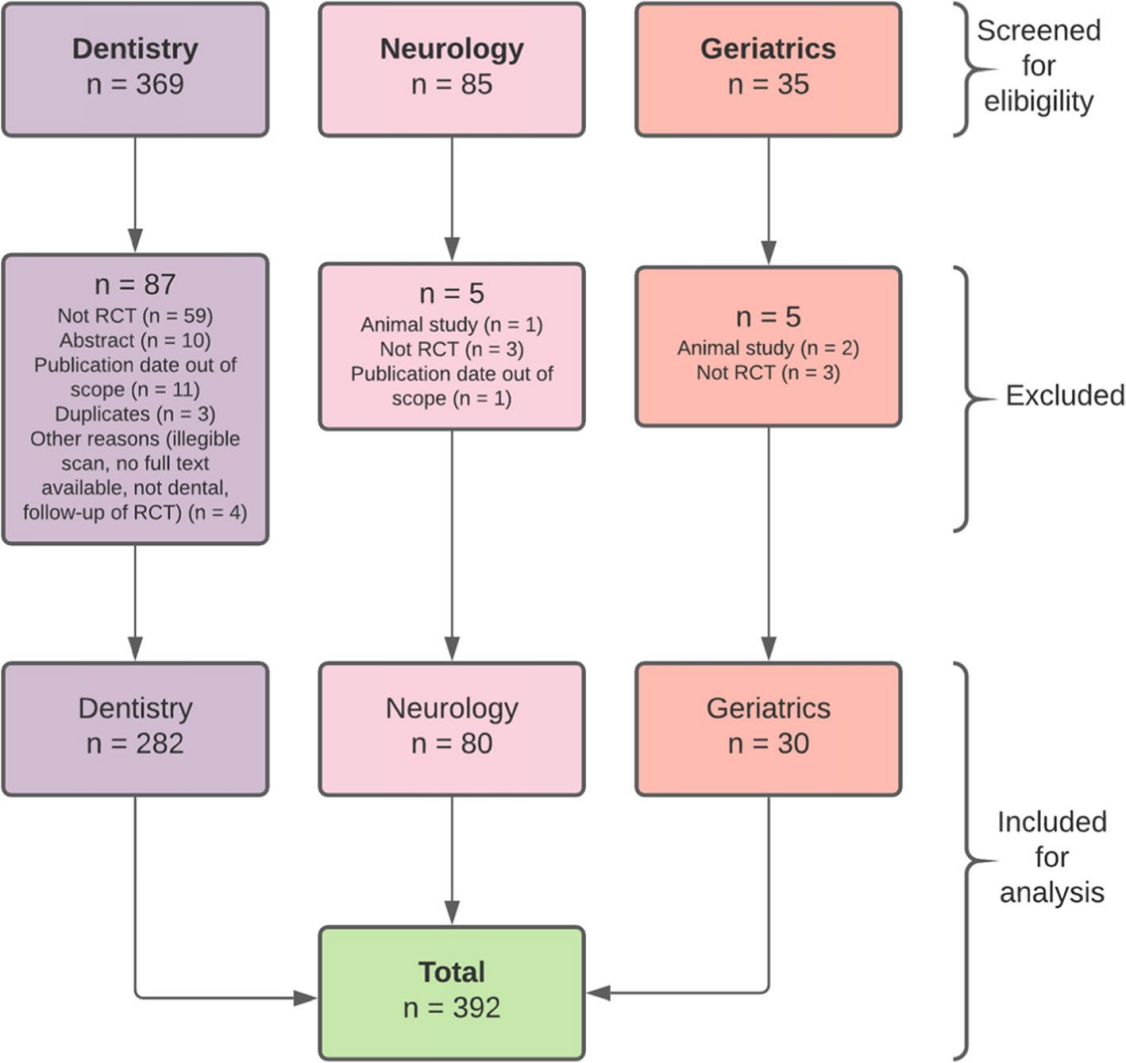
Flow diagram of study selection. Figure prepared by the authors based on study results

A total of 489 records were found in the BADERI database and collected by hand searching. Figure 1 shows the flow diagram for the article selection process and the distribution by speciality. Dentistry accounts for 76% of all the articles screened in our study. We explain this preponderance because dentistry contains all the specialities within it, while geriatrics and neurology are medical subspecialties. After screening, studies were excluded mainly due to not being randomised, only reporting an abstract, and being published in a year out of this study's scope. Finally, for analysis, a total of 392 articles for the period 1990 to 2018 were included as follows: 282 for dentistry, 80 for neurology, and 30 for geriatrics.

Sixty-nine journals were included in the study, corresponding to seven countries of South America, one country of North America (Mexico), and one country from Europe (Spain) that also had the largest number of journals (n = 24). Table 2 shows the distribution of journals and articles by country and by clinical field. Of the total of 392 RCTs included for analysis, 88 were published in English (81 in dentistry and 7 in neurology), 1 in Portuguese (dentistry), and 303 in Spanish (200 in dentistry, 73 in neurology, and 30 in geriatrics). The complete list of journals is provided as supplementary material, with years of publication, country, and impact factor [34]. Of the 69 journals, only four have an impact factor, and only two have an impact factor over 1.

**Table 2:** Distribution of journals and included RCTs by country and clinical field

Country	Dentistry		Neurology		Geriatrics	
	**Journals (N)**	**RCT (N)**	**Journals (N)**	**RCT (N)**	**Journals (N)**	**RCT (N)**
Argentina	2	6	1	2	1	1
Chile	8	62	2	4	0	0
Colombia	7	24	0	0	0	0
Ecuador	0	0	1	4	0	0
Mexico	8	19	3	22	0	0
Peru	5	16	0	0	0	0
Spain	16	143	6	47	2	29
Uruguay	1	1	0	0	0	0
Venezuela	5	11	1	1	0	0
	**52**	**282**	**14**	**80**	**3**	**30**

### Assessment of the quality of reporting

We assessed the quality of reporting for each included RCT by calculating a mean compliance score for the 23 CONSORT items that we included in this study. We also calculated each item's percentage of adherence across all RCTs. There are no missing data for this study, so all calculations were done on the 392 RCTs.

**Fig. 2:**
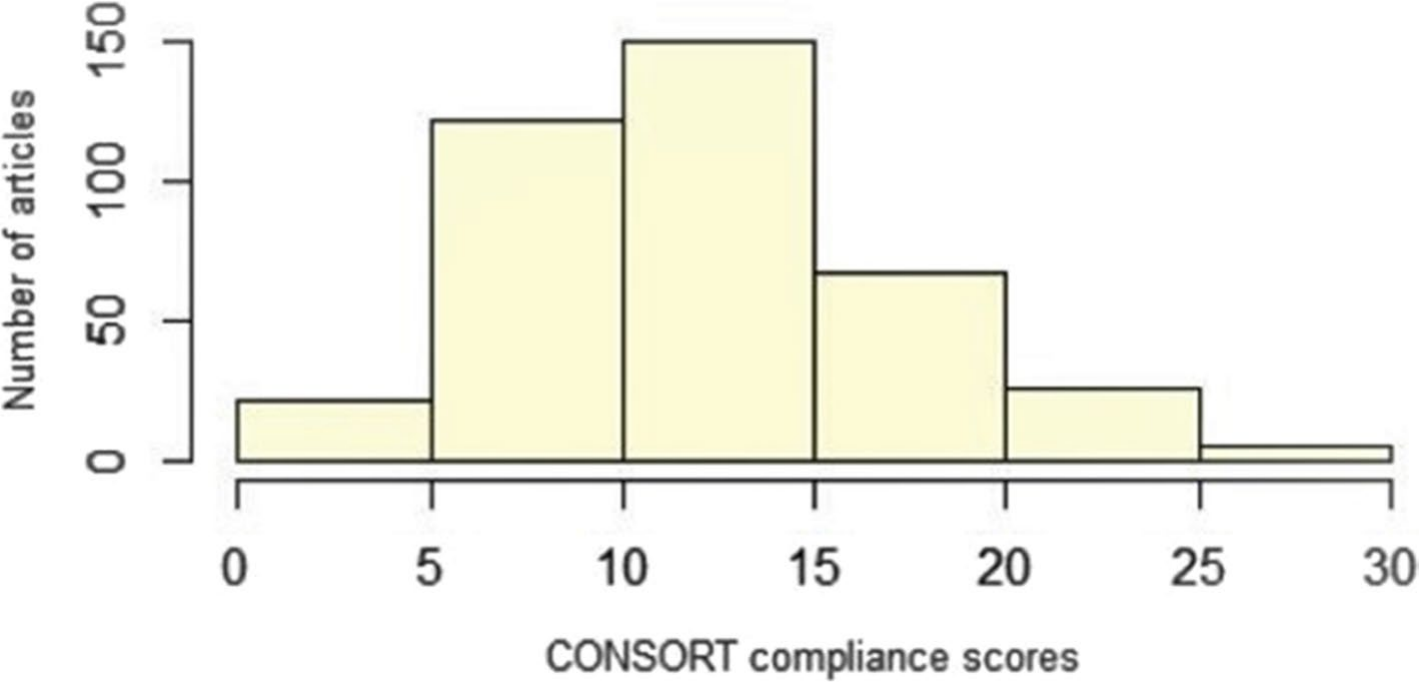
Distribution of CONSORT compliance scores (*n* = 392)

We found that the overall compliance score for the CONSORT items included in this study for all 392 RCTs analysed was 12.6 on a scale with a maximum score of 34 (Table 3). Figure 2 shows the number of articles according to their mean CONSORT compliance score. No article achieved the highest score, and 8% of the 392 had scores ranging from 20 to 30, the latter being the highest score found in this study.

**Table 3:** Mean CONSORT compliance scores by country and clinical field. Score scale from 0 to 34 (n = 392)

Country	Dentistry mean ± SD (RCTs)	Neurology mean ± SD (RCTs)	Geriatrics mean ± SD (RCTs)	Average score mean ± SD	Overall average score mean ± SD
Argentina	12.8 ± 5.3 (6)	11.5 ± 5.0 (2)	9.0 (1)	12.1 ± 5.3	12.6 ± 5.1
Chile	13.7 ± 6.0 (62)	12.3 ± 3.4 (4)	-	13.6 ± 6.0	
Colombia	14.8 ± 5.0 (24)	-	-	14.8 ± 5.0	
Ecuador	-	12.3 ± 2.6 (4)	-	12.3 ± 2.6	
Mexico	10.4 ± 4.6 (19)	11.4 ± 5.5 (22)	-	10.9 ± 4.6	
Peru	10.1 ± 4.2 (16)	-	-	10.1 ± 4.2	
Spain	13.2 ± 5.0 (143)	11.5 ± 4.8 (47)	11.6 ± 6.0 (29)	12.7 ± 5.0	
Uruguay	6.0 (1)	-	-	6.0	
Venezuela	11.1 ± 4.5 (11)	17.0 (1)	-	11.1 ± 4.5	

Empty cells indicate that there were no RCTs included*SD *Standard deviation

The distribution of the mean score by country and by clinical field is shown in Table 3. The country with the highest score was Colombia, but it only published trials in dentistry. Spain has by far the highest number of RCTs in geriatrics and, except for Argentina, which has one, no other country reports randomised intervention studies in this field. No differences were found between individual countries or after comparing Spain to Latin America regarding the three clinical fields' overall compliance scores.

The quality of reporting improved slightly with time for all RCTs, but more significantly for dentistry, which improved 5.2 points between 1990 to 2010 versus the 2015 to 2018 period, a 15% increase (Table 4).

**Table 4:** Overall mean scores over three time periods and by clinical fields

Time period	No. of RCTs	Dentistry	Neurology	Geriatrics	Overall score
1990 to 2010	189	10.5 ± 4.2	11.4 ± 5.1	9.5 ± 4.8	10.7 ± 4.5
2011 to 2014	99	13.6 ± 5.0	11.6 ± 3.6	13.9 ± 5.9	13.3 ± 4.9
2015 to 2018	104	15.7 ± 4.9	13.4 ± 4.6	13.0 ± 7.3	15.3 ± 5.1

*SD* Standard deviation

**Fig. 3:**
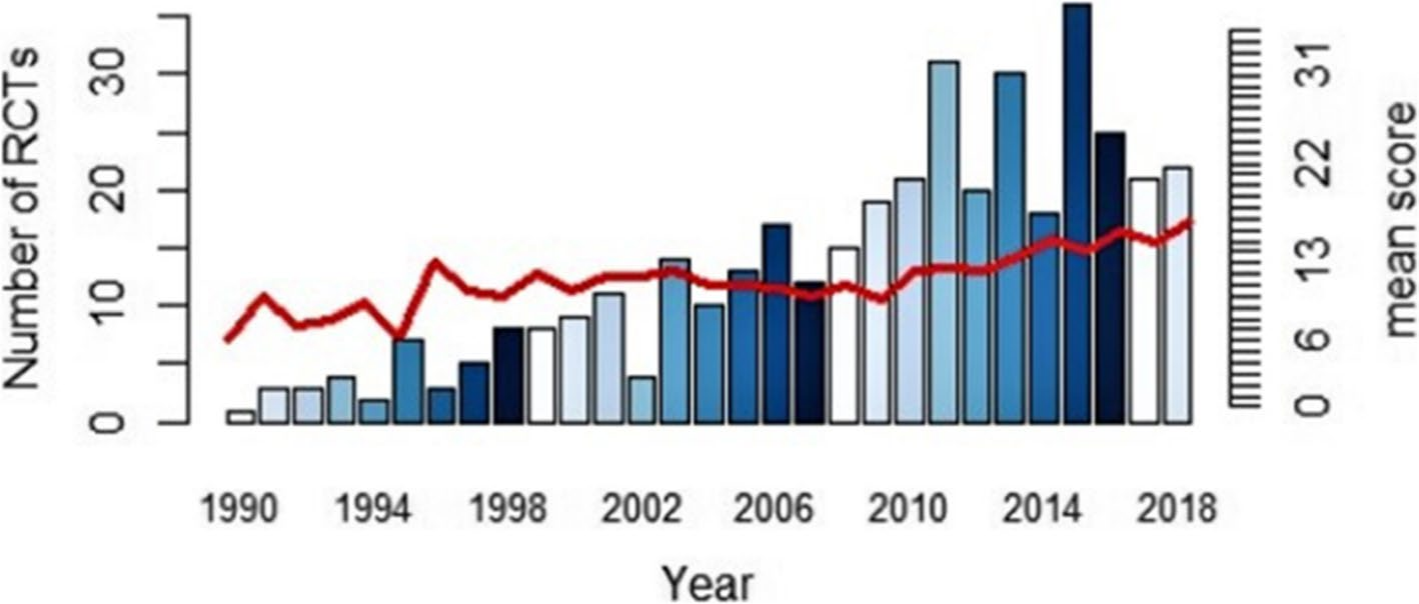
RCTs published and the mean score, by year

Another way of looking at our results is with a time trend analysis, as shown in Fig. 3. While roughly half of the RCTs (51.8%) were published after the last CONSORT statement was issued, the mean score improves only slightly, as mentioned above.

None of the articles achieved the maximum individual CONSORT item compliance score. The lowest overall compliance percentage was for item 10 (Randomisation implementation), and item 24 (Protocol registration), with a dismal 1% compliance across all included RCTs and regardless of country. Other very poorly reported items (less than 5% compliance) were item 14a (Recruitment), 17a (Outcomes and estimation), and 23 (Registration), all with an overall compliance proportion of 4%. The highest compliance percentages, over 70%, were found for items 5 (Interventions) with 86%, 4a (Participants) with 79%, and 1b (Abstract) with 70% (Table 5).

**Table 5:** The proportion of RCTs that comply with each CONSORT item included for analysis in the study, by region, and the overall compliance (*n* = 392)

Consort item and number			Latin America (%)	Spain (%)	All (%)
Title & abstract	1a	Title	16	30	24
	1b	Abstract	55	82	70
Methods	3a	Trial design	58	55	56
	4a	Participants	72	82	78
	4b	Settings	43	44	43
	5	Interventions	88	84	86
	6a	Outcomes	21	22	22
Randomisation	7a	Sample size	22	19	20
	8a	Sequence generation	28	35	32
	9	Allocation concealment	12	11	11
	10	Implementation	3	0	1
	11a	Blinding	24	18	21
	12a	Statistical methods	44	37	40
Results	13a	Participant flow	35	28	31
	13b	Losses & exclusions	20	19	19
	14a	Dates of recruitment	5	4	4
	15	Baseline data	29	39	34
	16	Numbers analysed	15	15	15
	17a	Outcomes & estimation	3	5	4
	19	Harms	26	29	28
Other information	23	Registration	2	5	4
	24	Protocol	0	1	1
	25	Funding	28	25	26

**Fig. 4:**
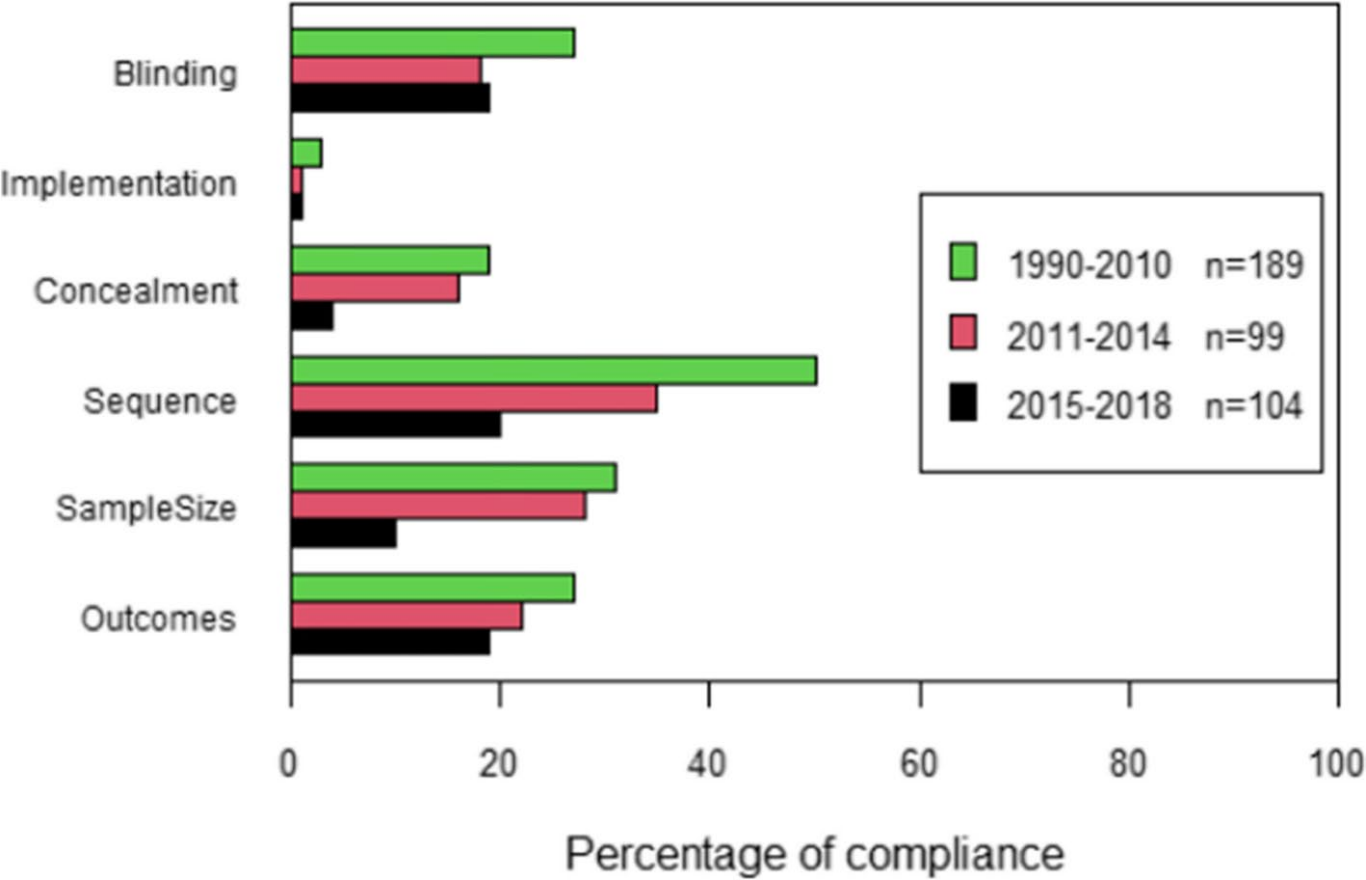
Overall compliance with CONSORT for six essential items, by periods

When analysing by period six essential items with a strong association with risk of bias in intervention trials, items 6a, 7a, 8a, 9, 10, and 11a (Outcomes, Sample size, Sequence generation, Allocation concealment, Implementation, and Blinding, respectively), no significant improvements were found even though there is a consistent tendency for greater overall compliance for each period, and especially for the years 2015 to 2018 (Fig. 4). Compliance was consistently under 50% for all these items for all the study periods.

### Pre-hoc sample size estimation and sample size

**Fig. 5:**
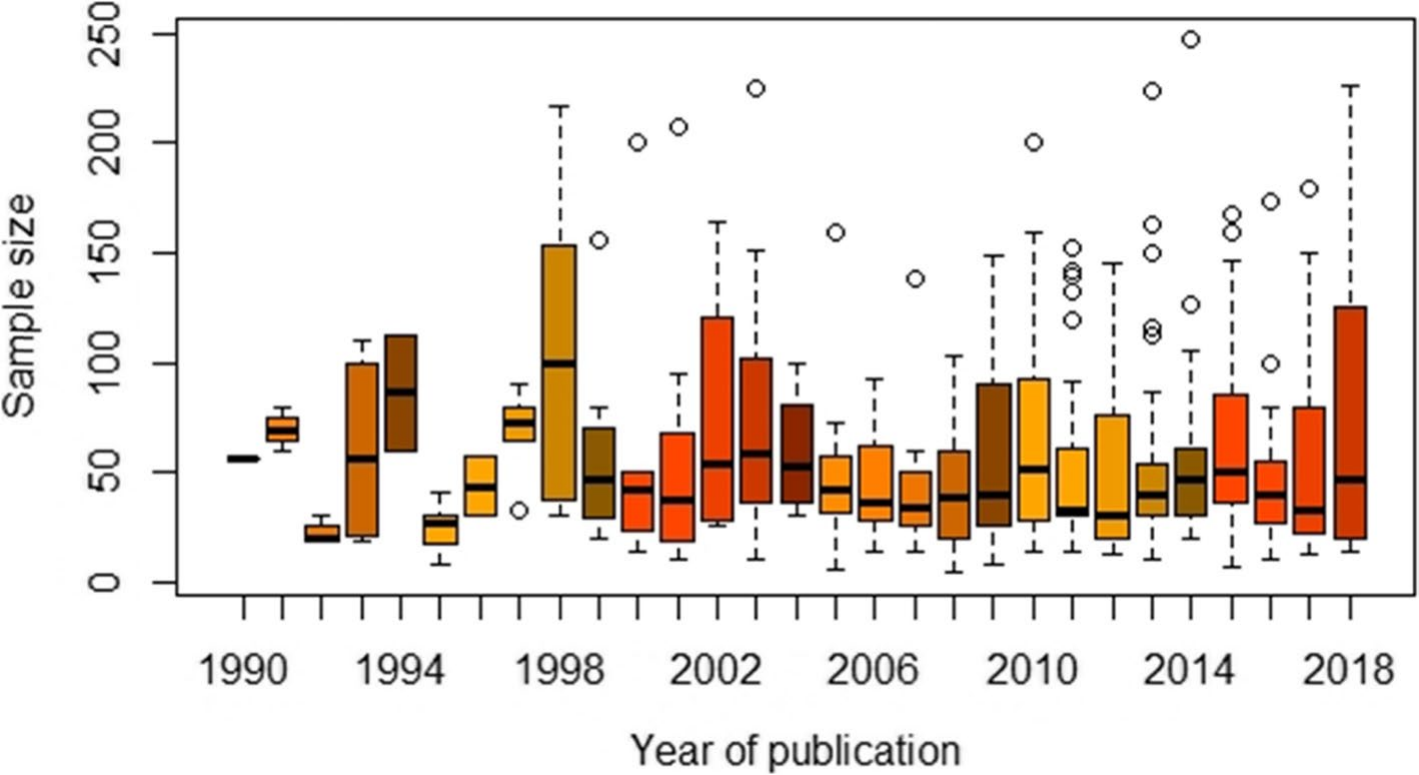
Sample size by year of publication (*n* = 377)

We extracted the sample size for all 392 RCTs. Figure 5 shows the sample size for 377 RCTs over time. Fifteen RCTs were excluded from the figure because the sample size was very large, over 300. Whilst this is not a CONSORT item, it is important to look at the sample size for the included studies as there seems to be a correlation between methodological quality and sample size. The median sample size for the whole period and the full roster of included RCTs was 46, with little variability over time. Seventy-nine articles (20%) reported having performed a sample size calculation before running the trial. Of these, only 29 trials (7%) recruited samples larger than or equal to 100 participants (50 per group).

## Discussion

After analysing 23 CONSORT items, our survey assessed the reporting quality of 392 randomised clinical trials from 69 journals based in Latin America and Spain from 1990 to 2018 for three clinical fields: dentistry, neurology, and geriatrics. We found overall poor compliance of 12.9 over a maximum score of 34 across all articles for the CONSORT items included in this study. Compliance was especially dismal for the items regarding Implementation, Protocol, Dates of recruitment, Outcomes and estimation, and Registration. The most compliant items were Interventions, Participants, and Abstract. Six items that are considered key for the risk of bias assessment were also poorly reported, consistently under 50% for the whole study period, although a slight improvement was seen for the latter years (2015 to 2018). Pre-hoc sample size calculation was reported only in 20% of the RCTs, and the sample size was small, as a norm, for the whole period.

### Comparison with other studies

Quality of reporting has been extensively studied in high-impact journals. It has been far less explored in developing countries or regions where grant-funded research is the exception rather than the norm. While funding and some aspects of publication ethics were extracted for this study, we do not report those results in this article.

The dismally low overall compliance rate found in the RCTs included in our study was not surprising. Other authors had already previously concluded that the quality of reporting is, in their words, "well below an acceptable level" [35], "deficient" [36], "variable and in need of improvement" [37] and that while poor reporting has decreased with time, "more could be done" [38]. This seemingly consensual appreciation of RCT quality of reporting in the literature emanating from wealthier countries may explain why we did not find significant differences between RCTs published in Latin American journals versus Spanish journals.

A systematic review of the completeness of reporting and journal endorsement found that RCT reporting quality was statistically significantly better in five key items in journals endorsing CONSORT than those that did not [31], recognising that the completeness of reporting of trials remains sub-optimal. Another similar study did not find conclusive evidence to support the relation between journal endorsement of reporting guidelines and the completeness of reporting [39]. We did not cover this variable in our analysis because many journals included in this study have been discontinued. Moreover, they were published only in hard copy, making it difficult to know the editorial policies they had when the RCTs were published, especially in light of the low number of RCTs that each journal publishes over the years.

Several cross-sectional or retrospective systematic surveys have been done in dentistry to assess compliance with the CONSORT statement [40–45]. Consistent with our findings, none of them reports optimal compliance. Luguercio et al. reviewed the literature to evaluate RCTs on bleaching and included 185 RCTs [40]. The most poorly reported items in this study were protocol, flow chart, allocation concealment, and sample size calculation, with 80% of the included RCTs scoring zero. This study's overall CONSORT compliance was only 16.7 ± 5.4 points over a maximum score of 32, while ours was 12.9 over 34. Another study explored compliance with CONSORT of RCT abstracts in four major orthodontics journals for a more recent period than ours, from 2012 to 2017. It concluded that several CONSORT extension items for abstracts were poorly reported, and the best-reported item was having a structured abstract [43]. While practically none of the journals in our study have an impact factor, our findings are consistent with this study because one of the items most frequently reported was a structured abstract, albeit with only 70% compliance. Borrelli et al. used the impact factor report for 2016 to identify craniofacial surgery journals and assess RCT compliance with the CONSORT statement [45]. Like the previous study that only included journals with an impact factor, this study also found compliance with CONSORT to be 56% (minimum 33%, maximum 94%) of applicable items reported. Several methods items were also lacking in compliance. Another study evaluated the RCT quality of reporting in the leading neurosurgical journals and three leading general medical journals [46]. This study found that the compliance score for the speciality journals was lower than the general medical journals. Consequently, even while these three studies only included journals with an impact factor, the included RCTs' compliance scores were also low, although higher than our overall compliance score, with room for improvement.

A few studies in neurology have assessed overall compliance with CONSORT guidelines in physical interventions for people with spinal cord injury [47], multiple sclerosis [48], and restless leg syndrome [49]. Only partial, suboptimal adherence was found in all these studies.

Abstract adherence to CONSORT extensions has also been assessed for cardiology [50], ophthalmology [51], anaesthesiology [52], periodontal disease [42], orthodontics [43], migraine and headache [53], gerontology and geriatrics [54], emergency medicine [55], surgery [56], and pain management [57]. Not one of these speciality studies has found completeness of reporting for abstracts and mostly agree that there is poor reporting, low compliance, room for improvement, and interventions are needed. These findings for abstract adherence to CONSORT extensions are aligned with the findings of the plethora of studies that assess full-article compliance, like ours.

One systematic review [58] and three studies have also explored journal endorsement of CONSORT [41, 55, 57], which we did not do. As stated previously, the time frame covered by our study is quite long, meaning that many of the journals are not published anymore, or the RCTs are only found in hard copy. Exploring journal policies at the time of publication is not feasible for our population of included journals. Still, it seems reasonable to infer that journal endorsement, while important, will not impact reporting guidelines adherence if not accompanied by journal enforcement. This much more stringent conduct has not been explored, to our knowledge.

Only 20% of our included RCTs reported a pre-hoc sample size calculation, which does not differ substantively from a cross-sectional bibliographic study that compared orthodontics to periodontics reporting [44]. This study found that an adequate sample size calculation was done in only 35.7% of their sample. Likewise, a comparative study of RCTs indexed in PubMed for 2000 and 2006 found that a median of 80 participants was recruited per trial for parallel-group trials [35], contrasting with our findings where most of the included studies had a median sample size of fewer than 50 participants per trial, except for four very early years. While a median of 80 may seem to be a small sample size, the difference of at least 30 participants per trial in our roster of RCTs may potentially impact the robustness of inferential analysis.

### Strengths and limitations

One of our study's strengths was that it included a large number of articles, and the three clinical fields that were represented were surveyed completely. It is unlikely that we missed articles. However, the choice of clinical fields was pragmatic as it was determined by the availability of articles already identified in the BADERI database and whether a reporting quality assessment had already been done or not. When we began our study, we considered reporting the findings separately for each clinical area. By the end of the identification and selection process, we were aware that the small number of RCTs published in neurology and geriatrics journals of Latin America and Spain would make any analysis meaningless. We recognise that we did not include journals from Brazil, thus leaving aside a potentially large number of RCTs. Having said this, we do not believe that their inclusion would have changed the results, given that the differences between Spain and Latin America as a whole are very minimal and all the journals are publishing largely non-compliant RCTs, regardless of the year or the country of publication.

Another limitation, even if design-dependent, was that we used the CONSORT 2010 checklist for the full roster of RCTs included, regardless of the year of publication. If older RCTs had followed the earlier versions of CONSORT, one would have expected that at least the most critical methodological items would have been correctly reported. Instead, this was not the case, and, even for key items, reporting is consistently lacking in our population of RCTs throughout the study period. Furthermore, items added to the 2010 version of CONSORT were not included in our data (some items b, for example).

We devised a data extraction form that excluded many of the more subjective or non-applicable items to achieve greater consistency in the decision of compliance that each reviewer made. However, many other items were categorised as compliant, partially compliant, or fully compliant, as per protocol [31]. Nonetheless, and to enhance comparability with other published studies on the quality of reporting for randomised trials, we dichotomised each item into compliant or non-compliant. This way, the inherent subjectivity of reviewer responses was minimised in the analysis. On the flip side, the lack of a standardised extraction form for the quality of reporting assessment purposes is a drawback as the CONSORT checklist was not devised for this. ‘Quality’ is a construct because it is something that we cannot see and directly measure [59], but ‘reporting’ we can measure with the checklists by assessing adherence to them. So, while some have cautioned against the use of reporting checklists as a tool to assess the quality of a paper even when they have not been validated for this [60], they are widely used for practical reasons to assess ‘quality of reporting’ [61]. Likewise, we fully agree with Germini et al. when they declare that the choice of method for a score calculation can be a matter of discussion. Like them, we also decided to rate all items equally to make our results comparable to previous reports [55].

Most likely, one of the main limitations of our study is that nine assessors did the review process. We conducted extensive training, calibration, pretesting, and pilot testing. We achieved a reasonable response consistency of at least 80% between reviewers, deviating from our original protocol that had aimed for 90%. We sought to overcome this inherent potential source of variability with a disciplined and well-conducted method to resolve discrepancies between reviewers as the senior methodologist identified them. Pairs of reviewers were asked to settle the differences by consensus, and, when not possible, a third assessor defined the final response. Furthermore, we added an extra level of quality control by having one of the co-authors check a random sample of 10% of the included RCTs. After contrasting the extraction from this random check to the one made by the reviewers, no significant inconsistencies were found.

### Implications

Many studies have been done on the quality of reporting of randomised clinical trials and compliance with the CONSORT guidelines. This research line was initiated in early 2000 [62–66] and has been reproduced by many across the globe, crossing many specialities. We approached three little-explored clinical fields to complement other studies on the quality of reporting and risk of bias of Latin American and Spanish RCTs [29, 67–70].

Not a single study included in the discussion of this paper reports high compliance rates. Conversely, there is a widespread consensus that poor reporting is the norm rather than the exception, with lots of room for improvement. We believe that there is no need to explore this problem further, given this broad concordance in the findings regardless of the journal quality or the impact factor.

As stated by Turner et al. in a systematic review that compared medical journals that endorse CONSORT Statement to journals that do not, journal endorsement may benefit the completeness of reporting of the RCTs they publish, but, according to these authors, the completeness of reporting remains suboptimal [58]. Thus, it is better to have journals endorsing reporting guidelines than not, but it appears insufficient. As Doug Altmann expressed in 2012 in the EQUATOR Scientific Symposium 2012 "ACT now: Accuracy, Completeness, and Transparency in health research reporting": endorsement without enforcement is useless (oral communication).

Consequently, the next step in this field of research is to promote interventions that will strengthen the methodological competencies of editors and peer reviewers, together with creating audit forms based on reporting guidelines such as the CONSORT Statement or STROBE (STrengthening the Reporting of OBservational studies in Epidemiology) that editors can use to assess how well their publications are complying with internationally set standards. In other words, we must stop exploring compliance and start exploring interventions and their effectiveness in the real-world medical literature.

Furthermore, a more focused approach is needed for speciality journals who seem to be lagging behind their equal-ranking general medical journals to strengthen their capabilities for complete, accurate and transparent reporting of clinical trials. Finally, journal editors of regions publishing in languages other than English, or journals based in the global South, should also pay special attention to researchers' needs in methodological training. The focus should be placed on understanding the importance of using reporting guidelines to plan the design and conduct of clinical research and to ensure robust enforcement of reporting guidelines during the peer review and editorial processes. Further research is mandatory in exploring the best interventions to strengthen competencies in these stages of scientific communication.

## Conclusions

According to our results, compliance with CONSORT guidelines is very poor in this population of RCTs. It is unclear what role the CONSORT statement has played in the slight improvement found in 2015 to 2018. More must be done to improve the completeness of RCT reporting in Latin America and Spain. The Iberoamerican region journals should become more involved in endorsing and enforcing adherence to the CONSORT guidelines. Further research should be focused on devising and testing interventions to enhance the uptake of reporting guidelines in speciality journals and journals without an impact factor.

## Data Availability

The dataset supporting this article's conclusions is available in the Figshare repository, [10.6084/m9.figshare.13187729] and https://figshare.com/account/home#/projects/92198.
